# The knowns and unknowns of cardiac autoimmunity in viral myocarditis

**DOI:** 10.1002/rmv.2478

**Published:** 2023-09-02

**Authors:** Kiruthiga Mone, Jay Reddy

**Affiliations:** School of Veterinary Medicine and Biomedical Sciences, University of Nebraska-Lincoln, Lincoln, Nebraska, USA

**Keywords:** autoantibodies, autoimmune myocarditis, autoreactive T cells, viral myocarditis

## Abstract

Myocarditis can result from various infectious and non-infectious causes that can lead to dilated cardiomyopathy (DCM) and heart failure. Among the infectious causes, viruses are commonly suspected. But the challenge is our inability to demonstrate infectious viral particles during clinical presentations, partly because by that point, the viruses would have damaged the tissues and be cleared by the immune system. Therefore, viral signatures such as viral nucleic acids and virus-reactive antibodies may be the only readouts pointing to viruses as potential primary triggers of DCM. Thus, it becomes hard to explain persistent inflammatory infiltrates that might occur in individuals affected with chronic myocarditis/DCM manifesting myocardial dysfunctions. In these circumstances, autoimmunity is suspected, and antibodies to various autoantigens have been demonstrated, suggesting that immune therapies to suppress the autoimmune responses may be necessary. From this perspective, we endeavoured to determine whether or not the known viral causes are associated with development of autoimmune responses to cardiac antigens that include both cardiotropic and non-cardiotropic viruses. If so, what their nature and significance are in developing chronic myocarditis resulting from viruses as primary triggers.

## 1 ∣ INTRODUCTION

The immune system evolves to fight infections. The immune response, which generally refers to the reaction of the immune system to microbial infections, requires the participation of various cell types that are grouped into innate and adaptive immune cells. The innate immune cells include phagocytes (neutrophils and monocytes/macrophages), dendritic cells (DCs), natural killer (NK) cells, gamma delta (γδ) T cells, NK-T cells, B1-cells, and innate lymphoid cells (ILCs). Of these, the roles of phagocytes and, to some degree, NK cells and ILCs have been described in both invertebrates and vertebrates, implying that these are the first responders to infections in all species.^1-4^ The adaptive immune cells, B cells and T cells, express distinct antigen receptors that are critical to prevent future attacks by generating memory responses, but their distinct roles have been described only in vertebrates.^5,6^ Nevertheless, immune cells are not expected to recognise self-antigens as foreign, and such faulty recognition can lead to autoimmunity. More than 80 autoimmune diseases (AIDs) are known to affect humans; they are the leading cause of death among young and middle-aged women and represent the third most common category of chronic diseases affecting approximately 14.7 to 23.5 million people (~8%) in the United States.^7-9^ Generally, viruses are common suspects in AIDs, and myocarditis is no exception. Myocarditis-affected individuals could recover, but a proportion of them can develop chronic myocarditis leading to dilated cardiomyopathy (DCM), in which autoimmunity is suspected. In this review, we present our views on the significance and implications of cardiac autoimmunity in the development of chronic myocarditis resulting from the viruses known to cause myocarditis that include both cardiotropic and non-cardiotropic viruses.

## 2 ∣ AIDs ARE DISORDERS OF THE ADAPTIVE IMMUNE SYSTEM

Unlike autoinflammatory diseases of the innate immune system,^10,11^ AIDs are primarily mediated by adaptive immune cells. Essentially, B and T cells mediate their effector functions independently or cooperatively in eliminating invading pathogens. Antibodies produced by B cells are critical for preventing infections caused by extracellular pathogens that include some viruses as long they are present outside the cells. Conversely, T cells are indispensable for eliminating established infections that might occur with intracellular pathogens, including viruses. Two subsets of T cells cooperatively eliminate intracellular pathogens. While exogenously acquired, phagosome-originated microbial antigens are presented to CD4 T cells in the context of Major Histocompatibility Complex (MHC) class II molecules; cytoplasmic proteins, importantly viral antigens generated endogenously, are presented to CD8 T cells through the class I pathway. However, their effector functions differ in that antigen-sensitised CD4 T cells facilitate their effects by producing cytokines, as opposed to effector CD8 T cells, termed cytotoxic T lymphocytes (CTLs), which kill the infected cells directly. The functionalities of these effector adaptive immune cells remain the same, whether directed towards self- or foreign antigens. Thus, when self-tolerance is broken, autoimmune responses can be mediated by autoantibodies, autoreactive T cells, or both. However, it isto be noted that detecting such responses does not necessarily mean that AIDs are manifested clinically,^12,13^ raising the question of how pathogenic autoimmune responses could be generated in those affected.

**FIGURE 1:**
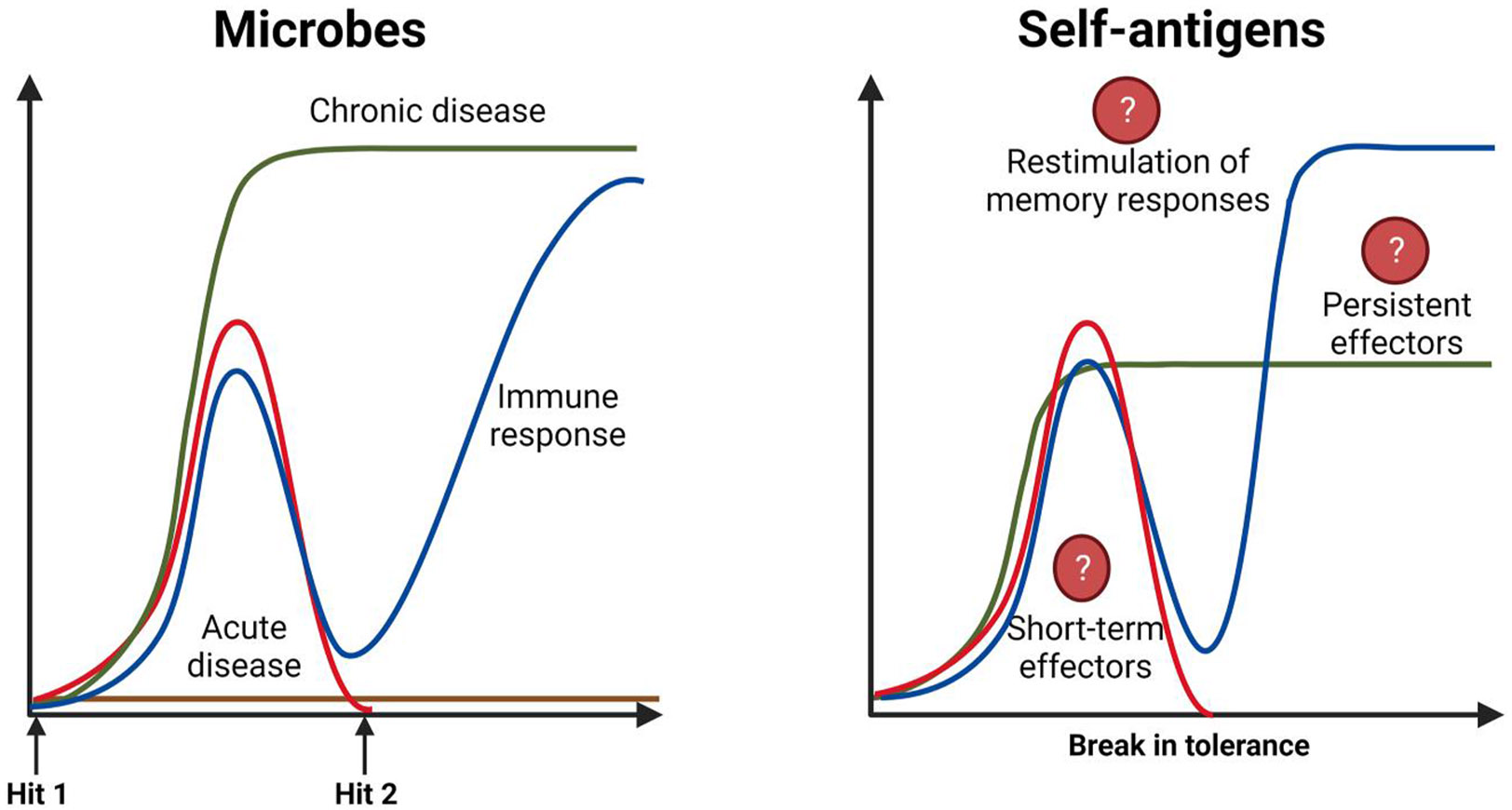
Hypothetical mechanisms of outcomes and immune responses between microbial and self-antigens. **Microbes**. Exposure to pathogenic microorganisms for the first time (termed hit 1) can lead to the induction of acute or chronic diseases, shown with red and green curves, respectively. As the immune system adapts to infection, acquired immune responses are set (blue curve), and upon re-exposure to the same pathogen (termed hit 2), memory cells swiftly react to prevent infections while strong memory responses continue to build in such future encounters. However, pathogens that induce chronic diseases may develop evasive mechanisms leading to their survival. **Self-antigens.** Although non-reactivity to self is one of the cardinal features of the adaptive immune system, under the conditions of a break in tolerance, the B cells and T cells can react to self-antigens (red arrow). But it is unknown whether such responses persist (green curve) or whether they recede over time, and if they do, it is unclear whether these responses can be continuously reactivated, leading to the induction of memory responses (blue curve). This figure was created using BioRender.com.

To illustrate this viewpoint, we have contrasted the patterns of immune responses between microbial and self-antigens, thus providing new insights into our understanding of AIDs (Figure 1). The healthy immune system can recognise millions of antigenic determinants arising from exposure to a vast array of microbes that could be pathogenic or non-pathogenic. Furthermore, humans could be exposed to millions of microbes or their particles that include fungal spores, virus-like and bacteria-like particles a day.^14^ Given the enormity of the surface areas available in the mucosal sites, especially the gut, estimated to be on the order of 400 m^2^ germs have great potential to enter our bodies, but their effects could vary.^15^

Upon exposure to pathogenic organisms during the first encounter (termed hit 1), two major outcomes, either acute or chronic disease states, can be expected (Figure 1, left panel). As the immune system adapts to an infection, the effector B cells and T cells facilitate the clearance of a pathogen, and the memory cells generated during the first encounter can swiftly react to the same offending agent in subsequent encounters (hit 2 and so on), giving no opportunity for pathogens to cause disease. When such a response is derailed or becomes ineffective, pathogens are able to survive, and chronicity sets in. Conversely, because exposure to non-pathogenic microorganisms leads to no disease, immune responses to such microbes would never be known or investigated. If these are the general patterns of anti-microbial responses, the means by which patterns of autoimmune responses could be superimposed on anti-microbial responses becomes a contentious issue. Unlike foreign (microbial) antigens, self-antigens are expressed widely and are abundantly available, yet the healthy immune system doesn't recognise these antigens as foreign. However, under the conditions of autoimmunity (discussed later), a break in self-tolerance can lead to the recognition of self-antigens as foreign, causing the generation of autoreactive B cells or T cells or both. Whether such responses follow the pattern of primary (short-term) anti-microbial effector responses or persist forever is hard to determine in real-life situations (Figure 1, right panel). Likewise, should memory responses to self-antigens be induced, they can potentially continue to contribute to tissue damage unless checked by immune modifiers.^16-19^ But, the critical question is what factors could contribute to the development of autoimmune responses.

## 3 ∣ FACTORS IMPLICATED IN THE OCCURRENCE OF AIDs

**FIGURE 2:**
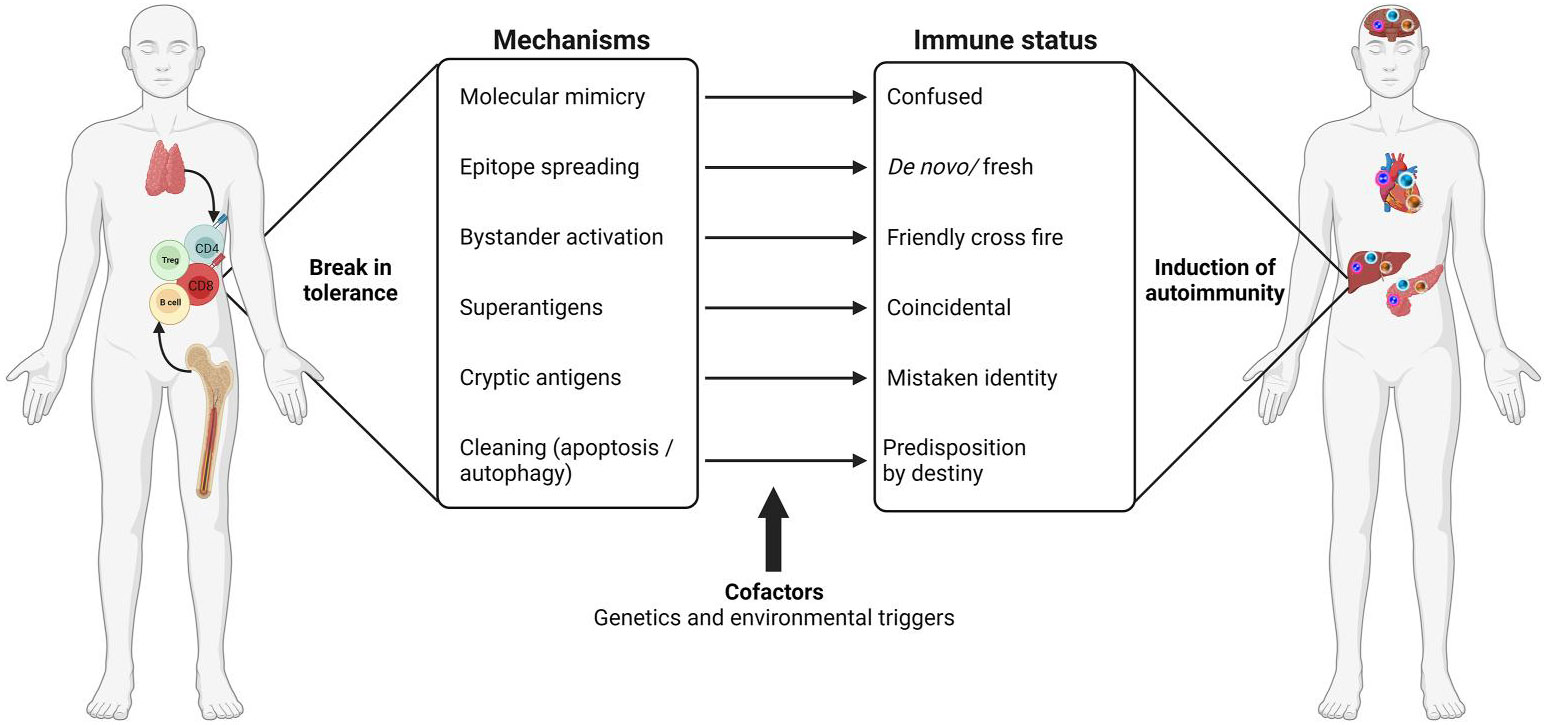
Generalised autoimmune mechanisms. Peripheral repertoires of healthy individuals may have autoreactive B cells, CD4 and CD8 T cells, but they may remain tolerant, and Treg cells occupy a central role in their maintenance of self-tolerance. However, in genetically susceptible individuals, self-tolerance can be broken under the influence of various environmental factors. While disease associations have been noted with various Major Histocompatibility Complex (MHC) and non-MHC genes, infectious and non-infectious agents can trigger autoimmune responses that may involve more than one mechanism in the genetically predisposed individuals. **First,** exposure to microbes carrying sequences similar to self-antigens can trigger autoimmunity by producing cross-reactive immune responses as a result of a confused immune state. **Second,** pathogens that have a tropism for specific tissues can lead to the generation of a *de novo*/fresh repertoire of autoreactive cells in response to the antigens released from damaged tissue through epitope spreading. **Third,** although autoreactive cells, if any, in healthy individuals are not expected to react to self-antigens, exposure to infections may stimulate antigen-presenting cells (APCs) to express costimulatory molecules needed to provoke autoreactive cells to become pathogenic as a result of bystander activation analogous to friendly cross-fire. **Fourth,** superantigens, by being polyclonal T cell activators, can activate self-reactive cells coincidentally if the autoreactive cells form a component of T cells targeted by superantigens. **Fifth,** tissue damage caused by exposure to drugs and chemicals can lead to the release of modified, cryptic antigens that can be seen by the immune system as foreign by mistake. **Sixth,** tissue-specific cell types in various organs may be continually replenished, and the dying or dead cells can be taken up by the resident APCs, leading to the presentation of self-antigens to autoreactive cells that might have pre-existed as a result of genetic predisposition. In all these scenarios, under the right conditions (signal-1, antigen; and signal-2, costimulatory/inflammatory cytokines), autoreactive cells can become pathogenic, causing tissue destruction as might happen in various organs such as the heart, liver, pancreas, and brain, among others. This figure was created using BioRender.com.

The peripheral repertoires of healthy humans may contain a proportion of self-reactive B cells and T cells, but they remain tolerant. Detection of these cells does not normally signify any ongoing pathological processes. The successful maturation of lymphocytes by positive selection requires recognition of self-antigens, but with weak affinity in the bone marrow for B cells and in the thymus for T cells in the context of MHC molecules, it is not uncommon to detect self-reactive cells in the peripheral compartment, although it has been suggested that self-reactive T cells may play a beneficial role in immune homoeostasis.^20^ However, it has been demonstrated that lack of expression of self-antigens in the thymus can facilitate the escape of autoreactive T cells from central tolerance; cardiac myosin (Myhc) is one example relevant to viral myocarditis.^21-25^ Such an escape mechanism for myosin-specific T cells can be reversed by transgenic expression of myosin in the thymus.^25^ Thus, any faulty presentation of self-peptides by MHC molecules can potentially allow T cells bearing high-affinity T cell receptors (TCRs) to escape from central tolerance^26^; several disease associations have been made with various human leucocyte antigen (HLA) alleles.^27-29^ However, suppose the MHC molecules are the primary predisposing genetic elements. If so, concordance of AIDs occurring in twins would be expected to be 100%, but that is not the case,^30-35^ suggesting that non-MHC genes could also contribute to the occurrence of AIDs. The best-characterised non-MHC genes are *AIRE, FoxP3, FAS,* and *FAS-L,* among others.^36-38^ Furthermore, autoreactive cells are also checked from reacting to self-antigens in the periphery by mechanisms that involve anergy, activation-induced cell death, and regulatory T cells (Treg).^39^ Arguably, it may also be possible that continuous exposure to self-antigens is critical to maintaining the peripheral tolerance.^40-42^ In support of this notion, we and others have demonstrated constitutive expression of cardiac antigens such as Myhc and sarcoplasmic/endoplasmic reticulum Ca^2+^-ATPase (SER-CA2a) by the antigen-presenting cells (APCs) in myocarditis-susceptible mice, but the animals do not spontaneously develop myocarditis.^43,44^ Although genetic susceptibility remains an important predisposing factor, it is hard to explain why some individuals with no genetic defects develop AIDs, leading to a suggestion that non-genetic (environmental) factors are also critical, and may include exposure to pathogens, dysbiosis, geographical locations, sex hormones, etc.^13,45,46^ Mechanistically, a break in self-tolerance could involve multiple pathways (Figure 2). These include molecular mimicry, epitope spreading, bystander activation, the release of cryptic antigens, and activation by superantigens, including antigens derived from cells undergoing apoptosis or autophagy. Excellent reviews are available that describe these mechanisms in detail.^47-50^ All factors considered, should pathogenic, autoimmune responses be generated in viral myocarditis, it is critical to determine their nature and the extent to which cardiac autoimmunity could contribute to chronic myocarditis and its long-term sequel, DCM.

## 4 ∣ CARDIAC AUTOIMMUNITY IN VIRAL MYOCARDITIS

### 4.1 ∣ Definitions

Inflammation of the heart muscle layer, the myocardium, is termed myocarditis, and the diagnosis is made based on histological, immunological, and immunohistochemical parameters. Myocarditis has been identified as the third leading cause (6%) of cardiovascular deaths in young athletes, next only to coronary artery abnormalities (17%) and hypertrophic cardiomyopathy (36%).^51,52^ Myocarditis is a predominant cause of heart failure in children and young adolescents^53-55^ and has been linked to the cause of sudden death in young adults/athletes in up to 12% of cases.^55,56^ Reports indicate that a proportion 30% of those affected with myocarditis could develop DCM.^57,58^ Likewise, recent studies show that ~50% of clinical diagnoses of DCM involve immunohistochemically detectable myocarditis.^59-61^ Clinically, DCM can be regarded as an end-stage disease. Due to the lack of effective therapeutic options, approximately half of DCM patients undergo heart transplantations,^62^ and children with acute myocarditis have only a 60% likelihood of transplantation-free survival at 10 years post-diagnosis.^63^ The DCM disease process is defined by decreased fractional shortening or ejection fraction and increased left ventricular end-diastolic diameter, excluding any known cause of myocardial damage, but is usually associated with cardiomyocyte loss.^64^ However, if myocarditis is associated with cardiac dysfunction in DCM patients, the term *inflammatory cardiomyopathy* is used.^52,64^ In these individuals, if viruses are detected, the suggested description is *inflammatory viral cardiomyopathy*, but in the absence of inflammation, the disease process is described as *viral cardiomyopathy*.^65^

### 4.2 ∣ Viral causes

Aetiologically, viruses are common suspects in myocarditis and include a wide range of virus types: cardiotropic (Adenovirus, Enterovirus, and Echovirus); cardiotoxic (Hepatitis C Virus, Human Immunodeficiency Virus [HIV], Influenza, and Coronaviruses); vasculotropic (parvoB19); and lymphotropic (Cytomegalovirus, Epstein Barr Virus [EBV], and Human Herpes Virus-6 [HHV-6]) (Table 1).^200^ Pathologically, however, it may be hard to draw a clear distinction between them because many of these viruses can affect organs other than the heart. For example, disease associations have been made with almost all six serotypes of Coxsackie B virus (CVBs) in relation to the heart, pancreas, brain, lungs, skin, eyes, and liver, among others.^201,202^ Notably, myocarditis and/or pancreatitis could be caused by CVB1 to CVB5.^201,202^ Thus, systemic responses generated consequent to the tissue damage in multiple organs may be difficult to delineate organ-specifically, which may confound interpretations of the data. Conversely, cardiotropic viruses may cause relatively more tissue damage in the heart than in other organs, and the resulting myocarditis can lead to DCM, but the infectious virions may or may not be detected in them.^203^ The only diagnostic signatures used to implicate viruses as primary triggers of DCM may be serological (virus-reactive antibodies) and molecular (viral nucleic acids) measurements. Furthermore, the cardiotropic viruses reported (Adenovirus, Enterovirus and Echovirus) are not typical reactivation types of viruses, although isolated reports indicate such a possibility, as shown with the Adenovirus and CVBs.^204-209^ However, repeat exposure to the same viruses can potentially lead to fresh tissue destruction in immune-compromised individuals, but such a possibility is less likely in healthy individuals because memory responses generated from first exposure would prevent reinfections. However, experimentally, repeat infection with CVB3 led to cardiac dilatation without inflammatory infiltrates and appearance of anti-cardiac actin and HSP60 antibodies.^93,210^ Given these complex scenarios, a conceptual framework has been built to indicate that chronic myocarditis/DCM might result from events secondary to viral damage, implicating autoimmune theory as a likely possibility. Enteroviruses, Parvovirus B19 and Adenovirus are commonly associated with myocarditis, and of these, Adenovirus is found to be the leading cause of myocarditis in children.^52,66,211-213^ This notion has changed as other viruses, including HHV6, are also frequently detected in myocarditis patients.^211,213^ Since autoimmune theory has gained significant attention in describing myocardial dysfunctions, we have endeavoured to understand the extent of autoimmune signatures in each virus infection in humans and experimental animal models, and identified gaps in the understanding of significance of cardiac autoimmunity in viral myocarditis.

### 4.3 ∣ Evidence for autoimmunity in viral myocarditis in humans

**FIGURE 3:**
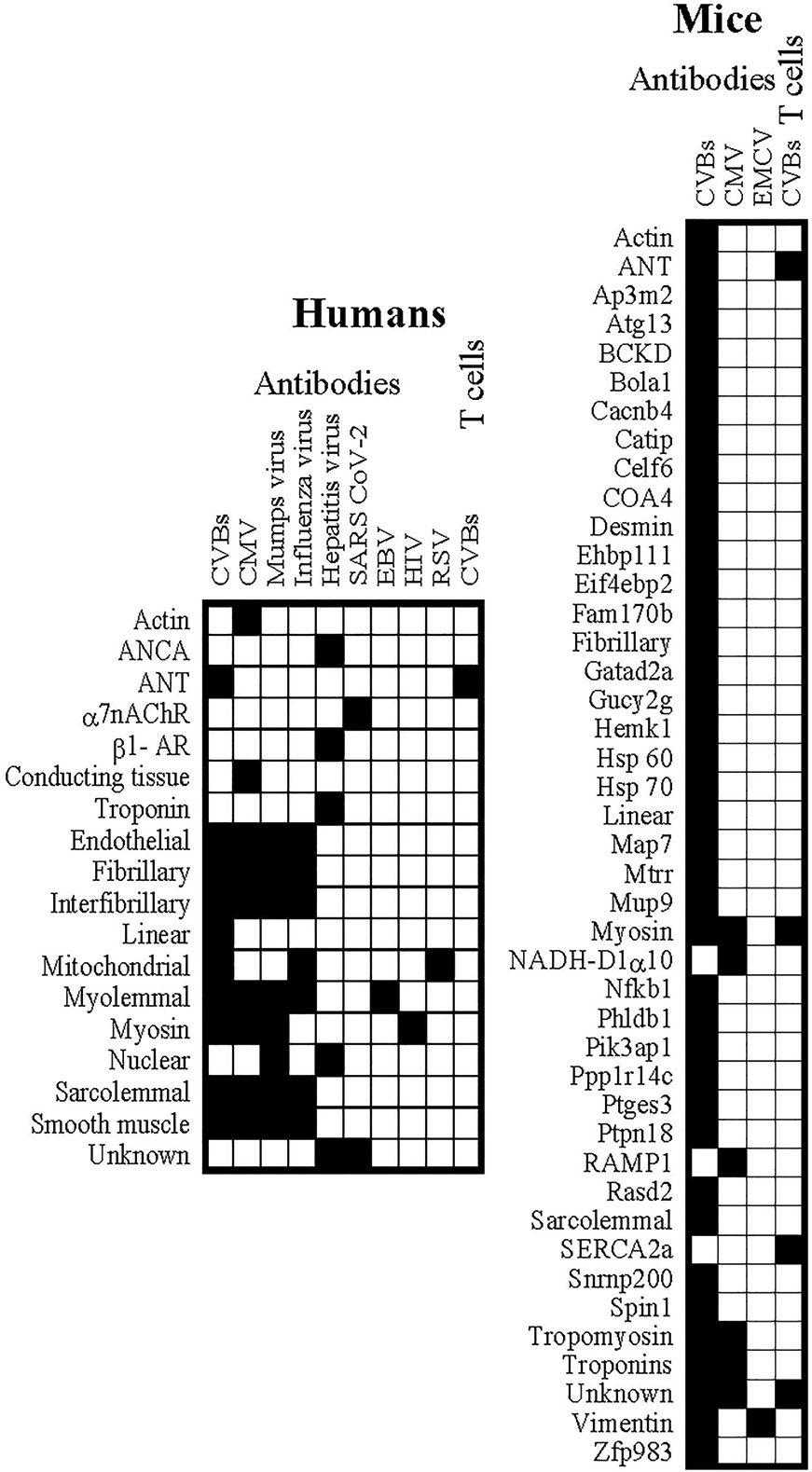
Heat maps display the detection of autoantibodies and autoreactive T cells in myocarditis associated with various virus infections in humans and mice. The left and right panels indicate autoantibodies detected in humans and mice, respectively, affected with various virus infections of viral myocarditis. The top legend represents viruses, and autoantibodies noted for various self-antigens are indicated on the left of each panel. Filled and empty squares represent the presence or absence, respectively, of antibodies noted in each virus infection. The last column in each panel represents detection of autoreactive T cells.

As indicated in Table 1, we noted that 40 different viruses can cause myocarditis in humans. Evidence for autoimmunity was shown by detection of antibodies to a variety of self-antigens in patients affected with Cytomegalovirus, Enteroviruses (mostly CVBs), Mumps virus, Influenza virus, and Hepatitis virus more frequently than others, such as Severe Acute Respiratory Syndrome Coronavirus-2 (SARS CoV-2), EBV, HIV, and Respiratory Syncytial Virus (RSV) (Table 1 and Figure 3, left panel). Autoantibodies were either not found or not investigated in individuals affected with infections caused by Adenovirus, Chikungunya virus, Dengue virus, Ebolavirus, Echovirus, Enterovirus-71, ParvovirusB19, HHV 6 and 7, HSV, Junin virus, Lassa fever virus, Lymphocytic Choriomeningitis Virus (LCMV), Measles, MERS CoV, Metapneumovirus, Monkeypox virus, Parainfluenza virus, Polio, Rabies, Reovirus, Rhinvovirus, Rotavirus, Rubella, SARS CoV, Vaccinia virus, Variola viruses, Varicella Zoster virus (VZV), West Nile virus, Yellow fever virus and Zika virus. Ironically, the role of autoreactive T cells has never been investigated in any of these virus infections except in CVB3 infection (Table 1, Figure 3 left panel).

### 4.4 ∣ Evidence for autoimmunity in viral myocarditis in laboratory animals

Experimentally, most of the viruses listed in Table 1 were found to induce myocarditis in mice. As in human myocarditis patients, autoantibodies have been documented in mice infected with CVBs and Cytomegalovirus and to a lesser extent with Encephalomyocarditis virus (EMCV) (Table 1 and Figure 3, right panel). However, no reports are available regarding the detection of autoantibodies in other viral causes, namely, Adenovirus, Chikungunya virus, Dengue virus, Ebolavirus, Echovirus, Enterovirus-71, ParvovirusB19, HHV 6 and 7, HSV, Junin virus, Lassa fever virus, LCMV, Measles, MERS CoV, Metapneumovirus, Monkeypox virus, Parainfluenza virus, Polio, Rabies, Reovirus, Rhinovirus, Rotavirus, Rubella, SARS CoV, Vaccinia virus, Variola viruses, VZV, West Nile virus, Yellow fever virus and Zika virus. In contrast to autoantibodies, investigations into the role of autoreactive T cells have been made only in CVB3 infection (Table 1, Figure 3 right panel).

Overall, by comparing the autoimmune signatures between humans and infection models, it may be fair to say that autoimmune responses, mainly production of autoantibodies, are more likely to be seen in myocarditis associated with infections caused by CVBs and Cytomegalovirus. Although such a trend has existed for Mumps virus, Influenza virus, and Hepatitis virus in humans, no reports are available for these viruses in laboratory animals (Table 1). However, the critical question to address is the nature/breadth of autoimmune responses noted in viral myocarditis, including antigen-specificity and myocarditogenicity.

### 4.5 ∣ Spectrum of autoimmune responses in viral myocarditis and their potential relationship to cardiac damage in humans

By analysing the autoantibody repertoires detected in myocarditis patients with various viral causes, it is evident that antibodies were noted for a wide range of self-antigens that can be specific or non-specific to heart tissue (Table 1 and Figure 3, left panel). The noteworthy cardiac-specific antibodies detected were troponins, that include cardiac Troponin I (cTNI), and Myhc in infections caused by Cytomegalovirus, CVBs, Hepatitis virus, and Mumps virus. Likewise, antibodies to β1-andregenic receptor (β1-AR), whose expression is preferentially noted in the heart among other tissues,^214^ were also noted in Hepatitis virus infection. Nevertheless, the majority of antibodies noted in most of the viral myocarditis patients were not specific to cardiac antigens. These include actin, ANCA, α7nAChR (potentially), BCKD, conducting tissues, endothelial, fibrillary, interfibrillary, linear, mitochondrial, myolemmal, nuclear, sarcolemmal, smooth muscle. Mechanistically, isolated reports are available to relate the pathogenic role of autoantibodies in myocarditis that may involve complement-dependent and/or complement-independent pathways. For example, the detection of heart-specific antibody complexes (e.g., Myhc) in heart tissues may indicate that heart-reactive antibodies might have caused tissue destruction,^96,99^ but raise the question how intracellularly located antigens can become visible to the antibodies. One possible scenario is that the intracellular proteins, upon release from viral damage to the heart, can lead to the formation of antibodies, which could then migrate to the heart and bind residual proteins present in the extracellular milieu by virtue of their tissue specificity. It is possible that the cardiomyocytes, under an inflammatory environment created by the host response to virus infection through cytokines such as IFN-γ, and IL-1β, intracellular antigens (eg., Myhc) can be potentially displayed on the surface of cardiomyocytes to be able to bind antibodies.^215^ Alternatively, viruses may cause damage to the heart epithelial tissue non-specifically, allowing the self-antigens to be exposed so antibodies can bind to them.^216^ Whether the heart-reactive antibodies can alter the functionalities of cardiomyocytes is unclear. Nonetheless, it has been shown that adenine nucleotide translocator (ANT) and CVB proteins can cross-react with each other in myocarditis and DCM patients,^100^ which can alter functionalities of ANT (e.g., energy metabolism).^101^ Similarly, antibodies to human Myhc could react with β1-AR that can lead to apoptosis of heart cells through proteinase kinase A (PKA) pathway,^217-219^ whereas anti-streptococcal M protein cytotoxic antibodies cross-reacting with human myosin can neutralise CVB3 and CVB4 or polio virus type I could result in autoimmune heart disease by cytotoxic reaction.^111^ Furthermore, reports indicate that β_1_AR-reactive antibodies can elevate the L-type Ca^2+^ current, leading to deleterious cardiac remodelling and DCM.^141^ Thus, it can be envisioned that heart-reactive antibodies could contribute to myocardial dysfunctions directly or indirectly. Although such detailed studies are lacking for other cardiac non-specific antigens, their participation in cardiac remodelling events cannot be ruled out. Alternatively, the formation of non-specific antibodies in myocarditis patients could result from tissue damage elsewhere in the body in response to systemic viral infection, making it difficult to consider them as potential biomarkers. All factors considered, it is generally expected that autoantibodies, if pathogenic, should transfer the disease in question―in this case, myocarditis/DCM. While such studies are not possible in human settings, isolated reports indicate that antibodies from myocarditis patients could transfer disease to severe combined immune deficiency (SCID) mice, supporting the idea that heart-reactive antibodies could have a pathogenic role.^102^ Likewise, antibodies to cTNI could induce cardiac dilatation and dysfunctions in programed cell death protein-1-deficient mice.^220^ Although, the role of T cells has not been examined in viral myocarditis patients per se, investigations from Cunningham's group revealed a role for Myhc-reactive T-helper 17 (Th17) cells in myocarditis and DCM patients that had no specific viral associations,^221^ but their pathogenic role is unclear. Likewise, peripheral blood leucocytes from patients with myocarditis could transfer disease to SCID mice supporting a role of T cells in the induction of myocarditis.^103^

### 4.6 ∣ Spectrum of autoimmune responses in viral myocarditis and their potential relationship to cardiac damage in laboratory animals in contrast to humans

The finding that most animal studies of viral myocarditis have been performed only in mice suggests that the mouse models are valuable tools for myocarditis research. Autoimmune responses have been investigated in various mouse strains mainly with CVBs and, to some degree, EMCV (both Enteroviruses) and Cytomegalovirus infections (Table 1 and Figure 3, right panel). By contrasting the autoantibody signatures of viral myocarditis in humans and animals, we made four observations that may or may not correlate with all virus infections. (1) Detection of antibodies to Myhc and ANT in both humans and mice in the context of Cytomegalovirus and Enterovirus infections suggests that they may have pathogenic significance (Figure 3, right panel). (2) Except for Vimentin-reactive antibodies in EMCV-infected mice, autoantibodies were not detected or investigated in other infections (Figure 3, right panel). (3) Some antibodies uniquely seen in mice but not in human myocarditis patients include Tropomyosin and Troponin in relation to Cytomegalovirus and Enterovirus infections, as well as branched-chain alpha-ketoacid dehydrogenase complex (BCKD)-reactivity in the latter (Figure 3, right panel). (4) Some degree of correlation exists between humans and mice with antibody reactivity to actin in myocarditis, although its detection was noted in infections caused by different viruses in mice (Cytomegalovirus in humans vs. Enteroviruses in mice) (Figure 3, right panel).

Furthermore, most animal studies have been limited to the detection of autoantibodies, and determination of their pathogenic role is reported rarely, but this may vary between mouse strains (Table 1). For example, the complement-depletion using the Cobra venom altered CVB3 myocarditis in DBA mice suggesting that heart-reactive antibodies might be responsible for myocardial damage. However, such a treatment did not alter the disease progression in Balb/c mice implying that the cellular immunity may be a contributing factor for CVB3 myocarditis in Balb/c mice.^125^ It is possible that the autoantibodies may not have a pathogenic role, but they could be regarded as biomarkers. In support of this proposition, antibodies to muscarinic M2 acetylcholine receptor, β1-AR, cTNI and BCKD have been detected in idiopathic DCM patients with no viral associations.^222-226^ An alternative interpretation could be that the breadth of autoantibodies might not have been characterised fully enough to determine their pathological significance. In that direction, we made efforts to use Phage ImmunoPrecipitation Sequencing (PhIP-Seq) to comprehensively analyse the autoantibody repertoire in the CVB3 myocarditis model in A/J mice, leading us to detect antibodies to 26 proteins that were not previously reported (Table 1).^110^ Furthermore, antibody reactivity patterns were similar in both CVB3 and CVB4 infected groups, but not in Influenza virus infection, indicating that multiple CVB infections can lead to the formation of similar autoantibodies. Ironically, however, the PhIP-Seq analysis did not consistently reveal antibodies for some of the commonly reported antigens, such as Myhc, and Troponins, previously reported by conventional methods in various virus infections (Table 1). Such discrepancies may reflect variations in the sensitivity of the assays used. Nevertheless, the finding that the autoantibodies reported in humans and mice belonged to IgG isotypes suggests a role for autoreactive CD4 helper T cells, as their help via cytokines is indispensable for isotype switching.^73,112,227^

Using serological analysis of recombinant cDNA expression libraries technology, T cell responses, including antibody reactivity to Myhc, were shown in Balb/c mice infected with MCMV and CVB3, potentially resulting from epitope spreading, but their pathogenic role was unknown.^74^ In our studies, to comprehensively address the role of antigen-specific T cells in CVB3 pathogenesis, we created MHC class II tetramers and dextramers (new version of tetramers) for five antigens, namely ANT, BCKD, β1AR, Myhc 334–352, and SERCA2a 971–990.^104,113^ First, we addressed the molecular mimicry hypothesis, but found no evidence for the appearance of cross-reactive T cells to ANT, β_1_AR, BCKDk, SERCA2a, and TNI that had sequences mimicking CVB3 proteins with similarities of 28%–47% as evaluated in the immunisation settings. We thus concluded that their cross-reactive T cells are unlikely to be generated in the infection setting.^104^ However, as we were enumerating the frequencies of antigen-specific T cells in CVB3 infection, we detected the appearance of T cells reacting to Myhc and SERCA2a in both the periphery (spleen and lymph nodes) and hearts. We also found that T cells reacting to both the antigens independently transferred disease in adoptive transfer experiments^104,113^ Unexpectedly, however, T cells specific to Myhc, SERCA2a, and ANT were also detected in the livers of CVB3-infected mice,^104^ suggesting that they can potentially recirculate and contribute to myocarditis in chronically infected animals, a possibility we are investigating currently. Furthermore, we discovered that Myhc 334–352 possesses epitope determinants for CD8 T cells, and by creating MHC class I tetramers, we demonstrated that Myhc-specific CD8 T cells infiltrate hearts of CVB-infected animals.^104,228^ Likewise, our recent investigations involving the determination of the immune landscape revealed a role for the cytotoxic nature of CD4 T cells that include Th17 and Treg cell subsets,^229^ and we are now analysing their antigen-specificity. We envision that a proportion of Myhc-specific CD4 T cells could contribute to CVB3 pathogenesis via cytotoxicity similar to CD8^+^ CTLs.

### 4.7 ∣ Major gaps in the understanding of the relevance of cardiac autoimmunity to heart tissue destruction in viral myocarditis

Accumulated literature suggests that autoimmunity forms a component of viral myocarditis in both human and animal studies. Since DCM may be an impending consequential event in individuals affected with myocarditis, detection of antibodies may correlate with DCM development, but conclusive proof is lacking as to the cause and effect relationships.

First, we emphasise the importance of distinguishing between specific and non-specific autoantibodies to cardiac antigens. The rationale for this proposition is that cardiac antibodies, by virtue of their specificity, are expected to cause damage primarily in hearts, but such a stringent expectation may not be relevant to non-specific antibodies. Nonetheless, in either situation, it is necessary to demonstrate that autoantibodies have a pathological significance. Unlike animal studies, where adoptive transfer models can be adopted to investigate the role of autoantibodies or autoreactive T cells, such investigations are impossible in human settings. Unfortunately, even in animal studies, only isolated reports are available to indicate that autoantibodies could transfer disease to naïve animals that may vary between mouse strains. This limitation further complicates the relatability of the observations made in inbred mouse strains to the outbred human population, because testing in one inbred mouse strain is genetically akin to testing in a single person. To overcome this limitation, two genetically diverse mouse lines have been developed, namely, collaborative cross (CC) and diversity outbred (DO) mice.^230,231^ Both CC and DO mice represent the genetic composition of eight different inbred mouse strains (five classic inbred and three wild-derived inbred).^230,231^ We recently used the DO mice to investigate the development of myocarditis in response to CVB3 infection, and, as expected, only a small percentage of DO mice showed heart infiltrates despite developing pancreatitis (manuscript in preparation). The use of such model systems may lead to the identification of quantitative trait loci that could be potentially relatable to humans, as demonstrated with SARS CoV-2.^232^ We plan to investigate these aspects in viral myocarditis induced with CVB3 in CC lines.

Second, infections with some of the cardiotropic viruses, such as CVBs, can cause tissue damage in non-cardiac organs―importantly, pancreas. An example is CVBs, where multiple serotypes (CVB1 to CVB5, with CVB3 as a prime candidate) could induce myocarditis and also pancreatitis. Therefore, evaluation of autoantibodies in surviving animals in order to relate their pathological significance to myocarditis may lead to misinterpretations, because, if formation of autoantibodies requires tissue damage for antigens to be released, they could be generated in response to both heart and pancreatic antigens. We encountered such an issue in our recent investigations with the PhIP-Seq analyses in the CVB3 infection model. Using the pathogenic Nancy strain of CVB3 that induces severe myocarditis and pancreatitis, and the E2 strain of CVB4 that primarily causes pancreatitis/insulitis, we noted similar antibody reactivity for select antigens in sera obtained from both infected groups.^110^ Additionally, tissue destruction resulting from systemic infection, as might occur in the hyperinflammatory syndrome associated with SARS CoV-2 infection, may lead to the development of autoantibodies to ubiquitously expressed antigens.^233^

Third, unlike liver, which has a remarkable capacity to regenerate, heart tissue, especially in adults, lacks regeneration capacity due to limited renewal of cardiomyocytes after injury, although reports suggest that cardiomyocytes can acquire regenerative potential by reentering the cell cycle.^234-236^ The cardiotropic enteroviruses (e.g., CVBs) primarily infect cardiomyocytes and cause extensive lysis leading to necrosis, whereas other viruses that may be found in the heart do not infect myocytes primarily due to a lack of receptors for their entry.^237,238^ Furthermore, host response to virus infections mediated by both innate and adaptive immune cells could also precipitate tissue damage through the production of inflammatory cytokines. While all these factors collectively contribute to heart tissue destruction, generation of autoimmune responses in cardiotropic virus infections is highly likely as reported due to the ability of the virus to replicate within cardiomyocytes and lyse them, allowing the intracellular proteins released from damaged myocytes to become autoimmune targets. This may be the reason for the detection of autoimmune responses to such proteins as Myhc and troponins,^99,124^ as well as mitochondrial proteins such as ANT and BCKD, among others.^104^ Thus, on one hand, it is possible that the cardiac antibodies may directly or indirectly alter myocardial functions as demonstrated with Myhc and ANT antibodies^101,239,240^; on the other, detection of autoantibodies may have no pathological significance. In both scenarios, cardiac remodelling events associated with myocardial dysfunctions could persist and be ascribed to a lack of regenerative capacity of cardiomyocytes.

Fourth, unlike autoantibodies, whose role can be relatively easily determined, it has been a challenge to investigate the role of cardiac reactive T cells in viral myocarditis, in part due to the lack of readily available tools such as MHC tetramers needed to enumerate the frequencies of antigen-specific T cells at a single cell level. Additionally, cytokines produced by different Th subsets (Th1, Th2, Th9, Th17, and Th22) could uniquely mediate their functionalities in different infection and autoimmune models,^241,242^ making it challenging to identify myocarditogenic cytokines in viral myocarditis. It is generally accepted that Th1 and Th17 cytokines may be critical to induce myocarditis, but Th17 cytokines appear to be indispensable for DCM development, as examined in autoimmune myocarditis models.^243-245^ Nonetheless, it is essential to provide evidence that the autoreactive CD4 or CD8 T cells generated in viral myocarditis, if any, are antigen-specific in order to relate their relevance to myocarditis pathogenesis.

**FIGURE 4:**
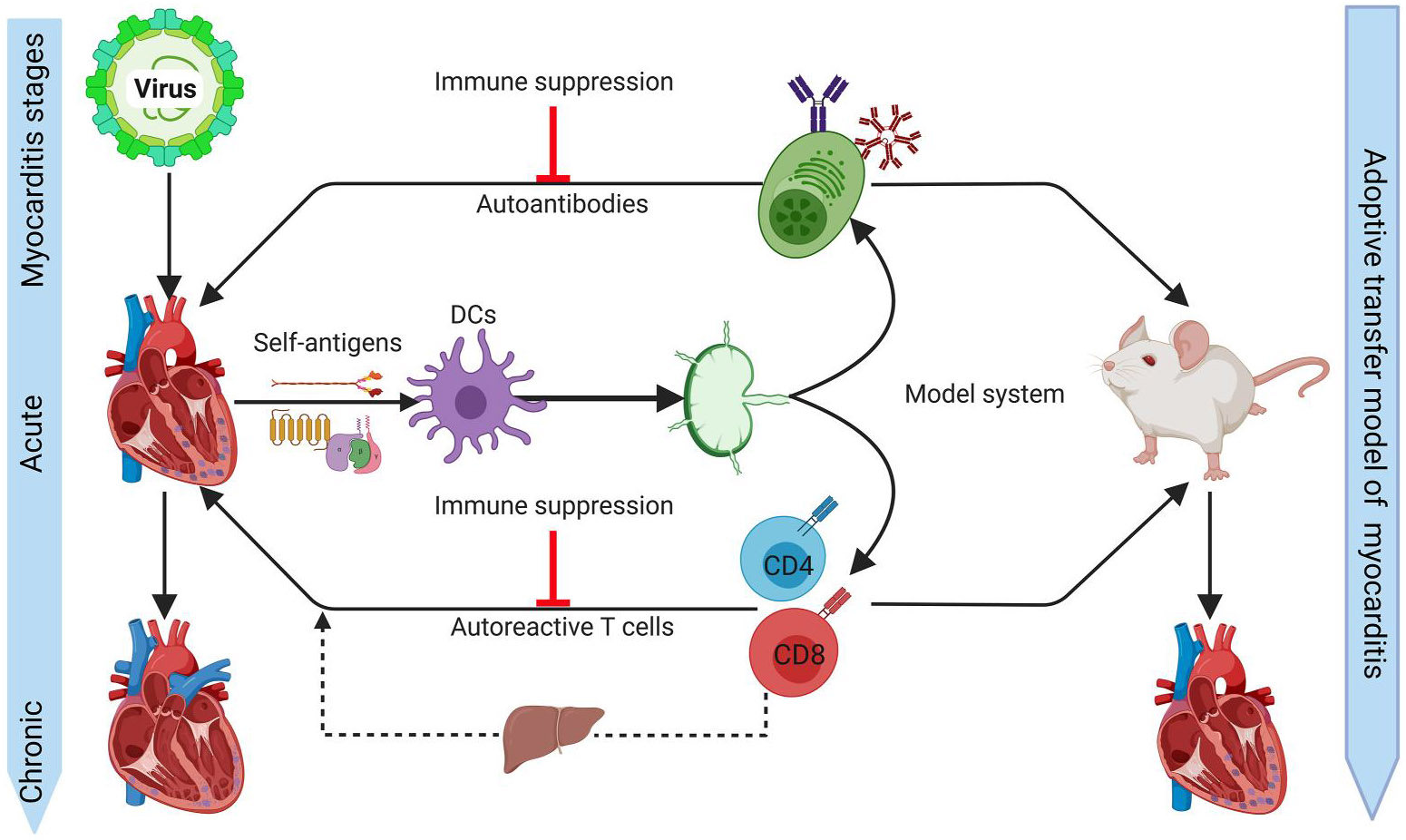
Autoimmune mechanisms of viral myocarditis in an experimental model system. Viruses can cause cardiac damage by direct injury leading to the development of acute myocarditis as indicated by the infiltration of immune cells that can lead to chronic myocarditis (left panel). During this process, resident dendritic cells (DCs) take up cardiac antigens released from damaged cardiac tissue leading to the generation of autoreactive B cells or CD4 and CD8 T cells by presenting antigens to the respective cell types in the draining lymph nodes that can recirculate back into the heart. A proportion of autoreactive T cells can reside in the liver with a potential for recirculation to the heart, and such emigrations can be potentially inhibited by immune suppressive strategies. Nonetheless, it is critical to confirm that the autoimmune responses are indeed pathogenic by using appropriate model systems (right panel). This figure was created using BioRender.com.

In that direction, evidence was provided by demonstrating that the CTLs harvested from CVB3-infected Babl/c mice could transfer disease in adoptive transfer models but antigen specificity was unknown.^105,126,127^ In our studies, we used a myocarditis model induced with CVB3 in which acute and chronic myocarditis phases occurring in continuum are well documented in myocarditis-susceptible A/J mice (Figure 4, left panel). Using MHC tetramers/dextramers, we demonstrated the appearance of pathogenic T cells with specificities for multiple antigens as evaluated in the adoptive transfer protocols (Figure 4, right panel). Unexpectedly, however, autoreactive T cells were found in the liver of infected animals at the same stage of infection, and whether the T cells parked in the liver can migrate to the heart is currently unknown. In these settings, we did not investigate whether autoantibodies to the corresponding antigens also appear; if they do so, determination of their pathogenicity in the adoptive transfer models is critical. Taken together, a conceptual framework can be built that the cardiac antigens released from the necrotic myocytes or those derived from the phagocytosed dying or dead myocytes can trigger the formation of autoreactive CD4 T cells that can reside in both lymphoid and non-lymphoid (e.g., liver) compartments with potential for them to migrate to the heart and contribute to chronic myocarditis under conditions of bystander activation in response to non-specific inflammatory stimuli. We are currently investigating this theory in CVB3 infection and adoptive transfer models of myocarditis (Figure 4). Proving this to be true may provide a basis to postulate a similar scenario in humans, because individuals affected with chronic myocarditis/DCM may have enteroviral signatures (virus-reactive antibodies and viral nucleic acids) without detectable infectious virions.^203^

**FIGURE 5:**
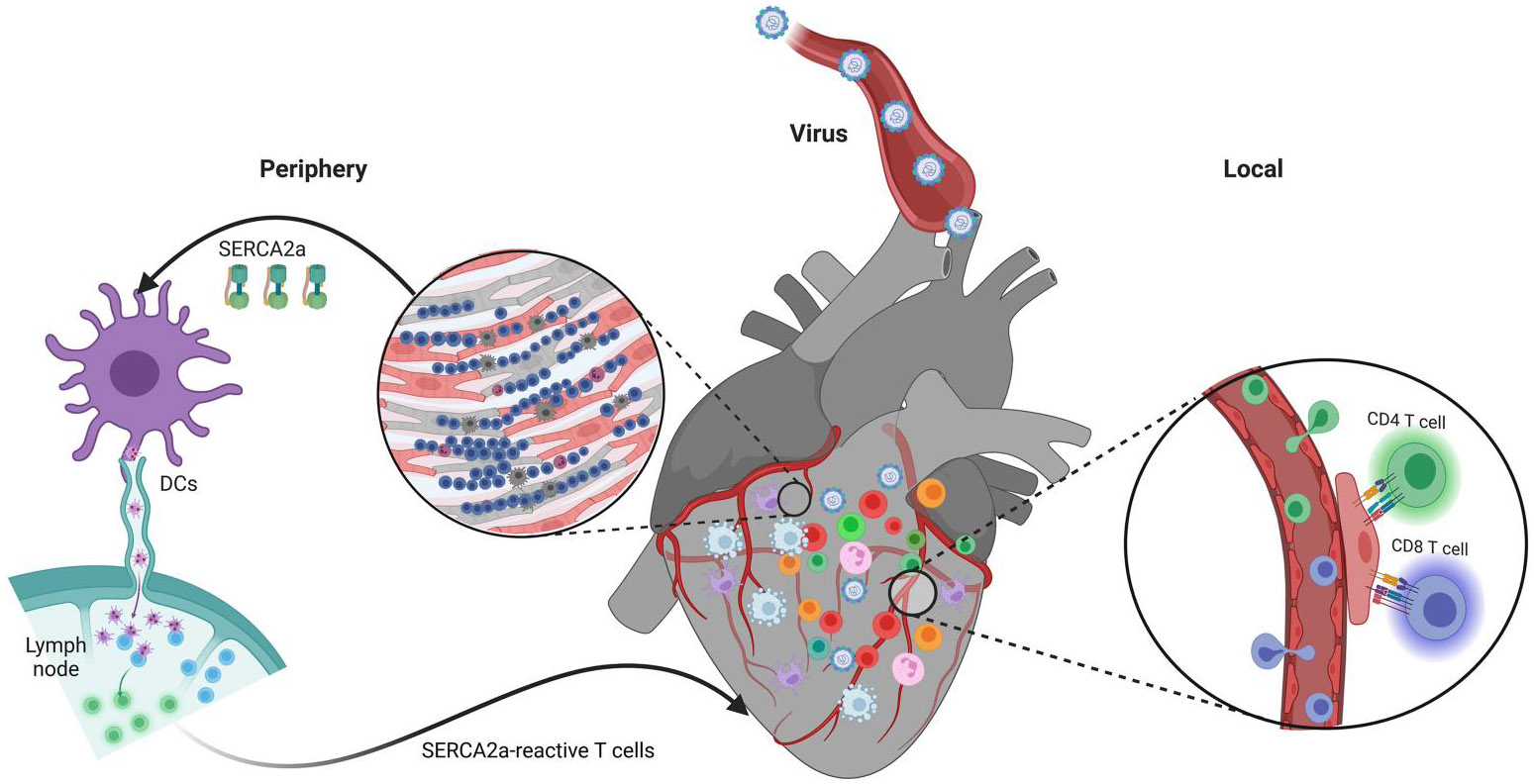
Potential mechanism of endothelial dysfunction in CVB3 myocarditis. Cardiac antigens such as SERCA2a released from myocardial damage could be taken up by the resident dendritic cells (DCs). They present antigens (eg., SERCA2a) in the draining lymph nodes leading to the generation of SERCA2a-reactive CD4 T cells that can activate endothelial cells (ECs), which facilitate the extravasation of cardiac-reactive T cells into the heart. During this process, the ECs can be killed by the cytotoxic function of SERCA2a-reactive CD8 T cells by an autoimmune reaction. This figure was created using BioRender.com.

We also propose that autoreactive T cells can cause endothelial damage in CVB3 myocarditis by demonstrating that SERCA2a 971–990 is constitutively expressed by APCs/endothelial cells (ECs) (Figure 5)^43^ and that SERCA2a-reactive T cells can induce EC death (unpublished observations). Of note, CVBs can infect ECs that express receptors for CVB in both human and animal models.^246-249^ Further, CVB induces expression of vascular endothelial-cadherin^250^ and intercellular gap junction proteins,^251^ as well as disruption of tight junction proteins in ECs.^252^ While CVB can survive for more than 260 days in ECs in vitro, virus-induced activation of ECs has been associated with altered permeability, increased expression of adhesion molecules (ICAM-1 and VCAM-1), DNA fragmentation/apoptosis, and cardiac fibrosis.^246,250,252-254^ Likewise, coronary EC dysfunction has been noted in idiopathic DCM patients^255^; microvascular spasms stimulated by EC damage have been noted in the pathogenesis of DCM^246,256^; and EC dysfunction has been recognised as an important predisposing factor for the development of vascular inflammation and coronary heart disease.^257-262^ Impaired EC function is noted in inflammatory cardiomyopathy patients with CVB persistence,^246,250,253,259,260,262^ and various viruses, including Enteroviruses, have been detected in atherosclerotic plaques, indicating that plaques are more susceptible to virus infection, which may facilitate myocardial infarction.^263-268^ While T cells from CVB-infected mice can lyse ECs by cytotoxicity^249,250,269^ and EC-reactive autoantibodies have been detected in viral myocarditis,^70,270,271^ target antigens are unknown. Our preliminary studies indicate that SERCA2a 971–990 possesses epitopes for both CD4 and CD8 T cells. Due to the promiscuity of SERCA2a expression in ECs, the SERCA2a-primed CD4 T cells generated in the periphery can activate ECs, facilitating extravasation of cardiac-reactive T cells into the heart (Figure 5). Thus, the cardiac-specific T cells generated in response to cardiotropic virus infections as a secondary event may have a significant role in the development of chronic myocarditis/DCM through multiple pathways.

### 4.8 ∣ Conclusions and implications

By critically analysing the literature available in human viral myocarditis patients and experimental infection models, we noted that autoantibodies have been extensively studied, but their relevance to the development of chronic myocarditis is insufficiently investigated to make conclusive interpretations. Since mechanistically addressing the role of autoimmune responses is not possible in human settings, the use of infection and adoptive transfer models in animal settings may help overcome this limitation, but observations made in the inbred mouse strains may or may not be relevant to the outbred human population. This is critical because, for example, genetic susceptibility has been well documented in mice in that the mouse strains with genetic background A (A/J and Balb/c) are susceptible to CVB3 myocarditis and develop a chronic course of the disease, whereas B strains (e.g., C57Bl/6) are relatively resistant and do not develop chronic myocarditis.^114,128,129^ Such a clear association with MHC alleles with viral myocarditis in humans has not been reported,^272^ although isolated reports indicate potential associations between HCV infection and hypertrophic cardiomyopathy in individuals bearing DBB1*0303 and DPB1*0901^273,274^; Coxsackievirus myocarditis and HLA antigens, A3, B40 and Cw2^275^; and EBV myocarditis with DR4 and DR13.^276^ Likewise, susceptibility to HCV-associated DCM was mapped to non-HLA gene locus from NFKBIL1 to MICA gene.^277^ Thus, the use of recently developed genetically diverse mouse strains such as CC and DO in infection studies may yield information regarding genetic susceptibility to viral myocarditis/DCM relatable to humans. Overall, in contrast to antibodies, the role of autoreactive T cells has rarely been studied in viral myocarditis patients, in part due to the lack of availability of appropriate tools. However, efforts have been made to analyse the role of cardiac-specific T cells in the mouse model of CVB3 infection by creation of MHC tetramers, leading to detection of autoreactive T cells specific to multiple cardiac antigens secondary to viral damage.^278^ Likewise, T cells can be parked in the peripheral lymphoid and non-lymphoid compartments during the post-viral phase of CVB3 infection with the possibility of recirculating back to hearts under conditions of bystander activation. Proving this to be true may provide a basis to envision a similar scenario in humans because individuals affected with chronic myocarditis may have viral signatures in the absence of detectable infections. Finally, among various experimental infections, CVB3 infection has remained the best model studied thus far. CVB pathogenesis exhibits an acute and chronic disease course occurring in the presence or absence of virus in the respective phases, and because it also resembles the disease features of DCM, it is an excellent model to address the virological and immunological mechanisms of viral myocarditis. However, when autoantibodies or autoreactive T cells are detected, it is critical to demonstrate their pathogenicity in adoptive transfer models since such studies are possible in laboratory animals; doing so may also create avenues to evaluate the efficacy of immune suppressive strategies (Figure 4). Failing to address the pathogenic role of autoimmune responses is unlikely to advance the myocarditis field, in the context of developing both new therapies and/or preventative strategies because viruses remain major disease triggers.

Translationally however, it is critical to stratify the DCM patients with or without virus origin in large clinical cohorts to determine the extent to which cardiac autoimmunity could be a contributing factor for disease pathogenesis. The rationale for this proposition is that some of the organ-specific autoimmune diseases such as multiple sclerosis are deemed autoimmune origin based on extensive clinical investigations leading to the use of disease-modifying therapies targeting autoimmunity.^279-284^ Unfortunately, such a guideline is not there yet or not routinely adopted for DCM patients although clinical trials have been carried out, but with mixed success.^285^ The investigations require analysis of autoantibody signatures for multiple cardiac antigens such as Myhc, cTNI, and SERCA2a in addition to β1-AR (although expressed in other organs)^286^ in combination with screening for viral signatures (antibodies or nucleic acids) using myocarditis virus panels. Screening for cardiac-reactive antibodies is imperative because of their specificity to heart and their pathogenic mechanisms may not necessarily involve inflammatory events. Rather alterations in the physiological functionalities of cardiomyocytes may be the key mechanisms. In support of this notion, administration of anti-cTNI antibodies have been shown to induce DCM in animal studies,^220,287,288^ whereas β1-AR antibodies could result in the prolonged activation of β1-adreno receptors leading to hyperadrenergic state resulting in apoptosis, fibrosis and heart failure.^289^ Similarly, antibodies to mitochondrial ANT or β1-AR could induce DCM by enhancing the calcium current.^288,290,291^ Additionally, cross reactive antibodies between self-antigens as shown with Myhc and β1-AR can cause apoptosis of myocytes.^217-219^ Recent investigations suggest that autoimmune calcium channelopathies may be relevant to DCM pathogenesis, and voltage gated calcium channels could be potential autoimmune targets in the development of DCM. For example, anti-α_1C_ Ca channel antibodies have been shown to be associated with cardiac electrical abnormalities, ventricular arrythmias and sudden death in DCM patients.^292,293^ Similarly, it is known that the DCM patients could have elevated antibodies to SERCA2a.^294,295^ Since SERCA2a being critical in calcium homoeostasis in the sarcoplasmic reticulum within cardiomyocytes, antibodies to SERCA2a may alter calcium cycle and disturb contractibility of heart muscle leading to heart failure.^295^ Finally, in addition to investigating the autoantibody signatures, determination of T cell responses for the corresponding proteins described above would be beneficial because of their critical role in the production of autoantibodies especially for protein antigens. Such an effort requires developing assays that are practically feasible, and one such assay may be ELISPOT and its variations.^296-299^ Accumulated literature indicate that cytokines produced by mainly Th1 and Th17 are proinflammatory^241^ and myocarditis and DCM patients could be associated with the production of Th17 cytokines.^221^ However, roles of cytokines produced by other Th subsets (Th9, Th22 and TFH) cannot be discounted including that of Th2 subset since their cytokines can influence antibody production similar to Th1 and Th17 cytokines, but pathways could be different. Overall, such investigations may provide a rationale or basis to explore the use of immune suppressive therapies in clinical settings. In that direction, newer modalities such as the use of peptides or aptamers to neutralise autoantibodies can be explored further,^289^ in combination with or without traditional approaches such as immunoadsorption, intravenous immunoglobulin therapy, biologics and selective immune suppressants targeting autoreactive B cells orT cells or both.^300-310^

## Figures and Tables

**TABLE 1:** Autoantibodies and autoreactive T cells detected in viral myocarditis.

Viruses	Antigens	Autoantibodies		Autoreactive T cells		Ref.
		Humans	Mice	Humans	Mice	
Adenovirus		Not reported		Not reported		66,67
Chikungunya virus		Not reported		Not reported		68,69
Cytomegalovirus (humans)/murine cytomegalovirus (mice)	Actin	✓	-	Not reported		70
	Endothelial	✓	-			70
	Fibrillary	✓	-			70
	Myolemmal	✓	-			70-72
	Sarcolemmal	✓	-			71,72
	Smooth muscle	✓	-			70-72
	Interfibrillary	✓	-			70-72
	Myosin	✓	✓ (Balb/c, C57Bl/6, C57Bl/10, Balb/c SM-LacZ)			73-79
	NADH-D1α10	-	✓ (Balb/c SM-LacZ)			74
	Conducting tissue	✓	-			70
	RAMP1	-	✓ (Balb/c SM-LacZ)			74
	Tropomyosin	-	✓ (Balb/c, C57Bl/10, Balb/c SM-LacZ)			73-75
	Troponin	-	✓ (Balb/c, C57Bl/10)			73,75
	Unknown	-	✓ (Balb/c, C57BL/10, C3H)			80,81
Dengue virus		Not reported		Not reported		82-85
Ebolavirus		Not reported		Not reported		86
Epstein-Barr virus	Myolemmal	✓	-	Not reported		72
Echovirus		Not reported		Not reported		87,88
EMCV	Vimentin	-	✓ (DBA/2)	Not reported		89
Enterovirus-71		Not reported		Not reported		90-92
CVBs	Actin	-	✓ (SWR/Ola, A/J)	-	-	93-95
	Endothelial	✓	-	-	-	70-72
	Fibrillary	✓	✓(A/J)	-	-	70-72,96
	Linear	-	✓(A/J)	-	-	96
	Mitochondrial	✓	-	-	-	70
	Myolemmal	✓	-	-	-	70-72,97,98
	ANT	✓	✓(A-strain mice, Balb/c, A/J, SCID, CVB3W, H3, H3-10A)	✓	✓(A/J)	75,98-109
	Ap3m2	-	✓(A/J)	-	-	110
	Sarcolemmal	✓	✓(A/J)	-	-	70-72,96-98
	Smooth muscle	✓	-	-	-	70-72
	Atg13	-	✓(A/J)	-	-	110
	BCKD	-	✓(A/J)	-	-	75,99,106,107
	Bola1	-	✓(A/J)	-	-	110
	Cacnb4	-	✓(A/J)	-	-	110
	Catip	-	✓(A/J)	-	-	110
	Celf6	-	✓(A/J)	-	-	110
	COA4	-	✓(A/J)	-	-	110
	Desmin	-	✓(SWR/Ola)	-	-	94
	Ehbp1l1	-	✓(A/J)	-	-	110
	Eif4ebp2	-	✓(A/J)	-	-	110
	Fam170b	-	✓(A/J)	-	-	110
	Gatad2a	-	✓(A/J)	-	-	110
	Gucy2g	-	✓(A/J)	-	-	110
	Hemk1	-	✓(A/J)	-	-	110
	Hsp 60	-	✓(SWR/Ola, A/J)	-	-	93,94
	Hsp 70	-	✓(SWR/Ola)	-	-	94
	Interfibrillary	✓	-	-	-	70-72
	Map7	-	✓(A/J)	-	-	110
	Mtrr	-	✓(A/J)	-	-	110
	Mup9	-	✓(A/J)	-	-	110
	Myosin	✓	✓(SWR/Ola, A/J, A.CA/SnJ, A.SW/SnJ, A.BY/SnJ, B10.A/SgSnJ, Balb/c, B10.PL/SgSf, B10.A/SgSf, CBA, CD-1, C3H/HeJ)		✓(A/J, Balb/c)	70,75,94,95,98,99,104,106,107,111-123
	Nfkb1	-	✓(A/J)	-	-	110
	Phldb1	-	✓(A/J)	-	-	110
	Pik3ap1	-	✓(A/J)	-	-	110
	Ppp1r14c	-	✓(A/J)	-	-	110
	Ptges3	-	✓(A/J)	-	-	110
	Ptpn18	-	✓(A/J)	-	-	110
	Rasd2	-	✓(A/J)	-	-	110
	SERCA2a	-	-	-	✓(A/J)	104,113
	Snrnp200	-	✓(A/J)	-	-	110
	Spin1	-	✓(A/J)	-	-	110
	Tropomyosin	-	✓(SWR/Ola, Balb/c, CBA)	-	-	94,121
	Troponin	-	✓(Balb/c)	-	-	124
	Unknown		✓(A.Sw/SnJ, Balb/c, DBA/2, A.BY/SnJ, A.SW/SnJ, A.CA/SnJ, C3H.NB/SnJ)		✓(Balb/c, CBA/J, Balb/c CUM)	105,125-136
	Vimentin	-	✓(Balb/c, CBA)	-	-	121
	Zfp983	-	✓(A/J)	-	-	110
Parvovirus B19		Not reported		Not reported		137-139
Hepatitis virus	Nuclear	✓	-	Not reported		140
	ANCA	✓	-			140
	β1- AR	✓	-			141,142
	Troponin	✓	-			143
	Unknown	✓	-			140,144
HHV 6 and 7		Not reported		Not reported		145-148
HIV	Myosin	✓	-	Not reported		149
HSV		Not reported		Not reported		150-153
Influenza	Endothelial	✓	-	Not reported		70-72
	Fibrillary	✓	-			71,72
	Mitochondrial	✓	-			70
	Myolemmal	✓	-			70-72
	Sarcolemmal	✓	-			70-72
	Smooth muscle	✓	-			71,72
	Interfibrillary	✓	-			71,72
Junin		Not reported		Not reported		154
Lassa fever virus		Not reported		Not reported		155-157
LCMV		Not reported		Not reported		158
Measles		Not reported		Not reported		159,160
MERS CoV		Not reported		Not reported		161,162
Metapneumovirus		Not reported		Not reported		163,164
Monkeypox virus		Not reported		Not reported		165-168
Mumps	Endothelial	✓	-	Not reported		70-72
	Fibrillary	✓	-			70-72
	Myolemmal	✓	-			70-72
	Nuclear	✓	-			70
	Sarcolemmal	✓	-			70-72
	Smooth muscle	✓	-			71,72
	Interfibrillary	✓	-			71,72
	Myosin	✓	-			70
Parainfluenza virus		Not reported		Not reported		169,170
Polio		Not reported		Not reported		171
Rabies		Not reported		Not reported		172-174
RSV	Mitochondrial-7	✓	-	Not reported		175
Reovirus		Not reported		Not reported		176-178
Rhinovirus		Not reported		Not reported		179,180
Rotavirus		Not reported		Not reported		181,182
Rubella		Not reported		Not reported		183,184
SARS CoV		Not reported		Not reported		162,185
SARS CoV-2	α7nAChR	✓	-	Not reported		186,187
	Unknown	✓	-			188
Vaccinia virus		Not reported		Not reported		189,190
Variola virus		Not reported		Not reported		191
VZV		Not reported		Not reported		192,193
West Nile virus		Not reported		Not reported		194,195
Yellow fever virus		Not reported		Not reported		196
Zika virus		Not reported		Not reported		197-199

## Data Availability

Data sharing is not applicable to this article as no new data were created or analysed in this study.

## Abbreviations:

α7nAChR: nicotinergic acetyl-choline receptor alpha7 subunit
β1-AR: beta-1 adrenergic receptor
γδ: gamma delta
AIDs: autoimmune diseases
ANCA: antineutrophil cytoplasm antibodies
ANT: adenine nucleotide translocator
Ap3m2: AP-3 complex subunit mu-2
APCs: antigen-presenting cells
Atg13: autophagy-related protein 13
BCKD: branched-chain alpha-ketoacid dehydrogenase complex
Bola1: BolA-like protein 1
Cacnb4: voltage-dependent L-type calcium channel subunit beta-4
Catip: ciliogenesis-associated TTC17-interacting protein (fragment)
CC: collaborative cross
Celf6: CUGBP Elav-like family member 6
COA4: cytochrome c oxidase assembly factor 4 homologue, mitochondrial
CTLs: cytotoxic T lymphocytes
cTNI: cardiac Troponin I
CVB: Coxsackie B virus
DC: dendritic cells
DCM: dilated cardiomyopathy
DO: diversity outbred
EBV: Epstein Barr Virus
ECs: endothelial cells
Ehbp1l1: EH domain-binding protein 1-like protein 1
Eif4ebp2: eukaryotic translation initiation factor 4E-binding protein 2
ELISPOT: enzyme-linked immunosorbent spot
EMCV: encephalomyocarditis virus
Fam170b: protein FAM170B
Gatad2a: transcriptional repressor p66 alpha
Gucy2g: guanylate cyclase 2G
Hemk1: HemK methyltransferase family member 1
HHV: Human Herpes Virus
HIV: Human Immunodeficiency Virus
HLA: human leucocyte antigen
Hsp: heat shock protein
HSV: Herpes Simplex Virus
ILCs: innate lymphoid cells
LCMV: Lymphocytic Choriomeningitis Virus
Map7: ensconsin
MERS CoV: Middle East Respiratory Syndrome Coronavirus
MHC: Major Histocompatibility Complex
MICA: Major Histocompatibility Complex class I chain-related protein A
Mtrr: methionine synthase reductase
Mup9: major urinary protein 9
Myhc: cardiac myosin
NADH-D1α10: NADH dehydrogenase (ubiquinone) 1 alpha subcomplex 10
Nfkb1: nuclear factor NF-kappa-B p105 subunit (fragment)
NFKBIL1: nuclear factor NF-kappa-B inhibitor-like protein 1
NK: natural killer
Phldb1: pleckstrin homology-like domain family B member 1
PhIP-Seq: Phage ImmunoPrecipitation Sequencing
Pik3ap1: phosphoinositide 3-kinase adaptor protein 1
PKA: proteinase kinase A
Ppp1r14c: protein phosphatase 1 regulatory subunit 14C
Ptges3: prostaglandin E synthase 3
Ptpn18: tyrosine-protein phosphatase non-receptor type 18
RAMP1: receptor activity modifying protein 1
Rasd2: GTP-binding protein Rhes
RSV: Respiratory Syncytial Virus
SARS CoV: Severe Acute Respiratory Syndrome Coronavirus
SARS CoV-2: Severe Acute Respiratory Syndrome Coronavirus-2
SCID: Severe Combined Immunodeficiency Disease
SERCA2a: sarcoplasmic/endoplasmic reticulum Ca2+ ATPase 2a
Snrnp200: U5 small nuclear ribonucleoprotein 200 kDa helicase
Spin1: spindlin-1
TCRs: T cell receptors
TFH: follicular helper T cells
Th: T helper cells
TNNC1: troponin C1
Treg: regulatory T cells
VZV: Varicella Zoster Virus
